# Severe reflux esophagitis and multiple congenital defects: A case report

**DOI:** 10.1097/MD.0000000000021758

**Published:** 2020-08-28

**Authors:** Jia-Yi Ma, Dan Wang, Zhao-Shen Li, Liang-Hao Hu

**Affiliations:** a Department of Gastroenterology, Gongli Hospital; b Department of Gastroenterology, Changhai Hospital, The Second Military Medical University, Shanghai, China.

**Keywords:** case report, congenital malformation, gastroesophageal reflux disease, whole exome sequencing

## Abstract

**Introduction:**

Gastroesophageal reflux disease is a common and troublesome condition. This paper reports a rare case of gastroesophageal reflux disease caused by ectopic biliary drainage accompanying the absence of a pyloric channel and duodenal bulb in a female patient with multiple underlying malformations.

**Patient concerns:**

A 24-year-old female presented with acid regurgitation and abdominal pain for one month. She was born two weeks premature and with blindness of the right eye. Cardiac murmur was detected in the physical examination.

**Diagnosis:**

Gastroendoscopy was performed, and a class D reflux esophagitis and ectopic papilla complicated with the absence of a pyloric channel and duodenal bulb were found. Doppler echocardiography further confirmed the defects of atrial and ventricular septa. Trio-based whole exome sequencing was performed on the proband and her family to find the potential association of multiple variations. However, no putative pathogenic mutations were found.

**Interventions:**

The patient received proton pump inhibitors and prokinetic treatment and underwent surgical repair of septal defects.

**Outcomes:**

The symptoms were quickly relieved, and the patient was kept stable upon follow-up.

**Conclusion:**

The combination of an absent pylorus and ectopic papilla is a rare cause of reflux esophagitis. Unusual gastrointestinal anatomical variations may be accompanied by other malformations. Though no remarkable mutation were detected in this case, sequencing is an efficient technique worth full consideration.

## Abstract 2

Supplemental Digital Content is available in the text

## 1 Introduction

Gastroesophageal reflux disease (GERD) is a common condition with prevalence rates varying from 8% to 33% according to geography and ethnic groups.^[1]^ Heartburn, regurgitation, epigastric pain, and sleep disturbance are the main symptoms that adversely affect an individual's day to day functioning and quality of life.^[2]^ Hiatus hernia, esophageal atresia, intestinal malrotation, omphalocele and gastroschisis are the common congenital defects that cause GERD.^[3]^ In this article, we report a rare case of GERD due to an anatomical variation of the papilla as well as the absence of pyloric channel and duodenal bulb, which concomitantly carry anomalies in the ocular and cardiovascular system.

## 2 Case presentation

**Figure 1:**
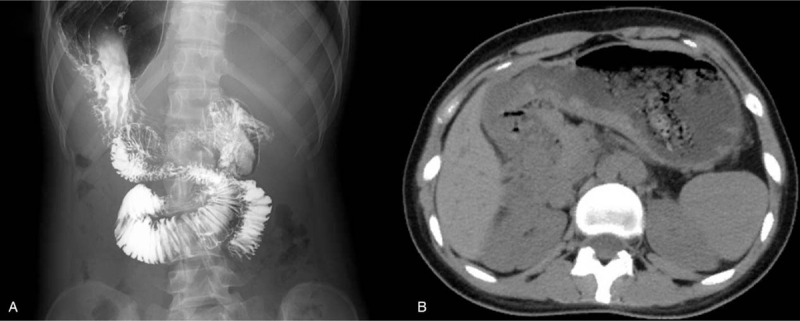
A, B Barium examination and CT scan revealed that the pyloric channel and duodenal bulb were absent. CT = computed tomography.

A 24-year-old girl was referred to the outpatient department because of one-month history of acid regurgitation and abdominal pain. The patient was born with an eyeball atrophy and blindness of the right eye but denied other concomitant diseases. She was two weeks premature. Her sister was delivered full-term and healthy. No drug has been applied in the past 3 months. Physical examination showed increased intensity of pulmonary second sound and systolic murmur at the 2nd to 4th intercostal space near the left sternal border. No positive abdominal sign was found. Upper gastrointestinal barium examination revealed that the folds of esophagus were continuous, while the wall was soft, and the peristalsis and evacuation were normal. No stenosis or filling defects were found. The stomach was low tension and hook-shaped, while the mucosal appearance was quite normal. Unexpectedly, the pylorus and duodenal bulb were invisible. Barium was going through the duodenal loop with a high speed. Abdominal computed tomography was further complemented, but no other malformations were found (Fig. 1A,B).

**Figure 2:**
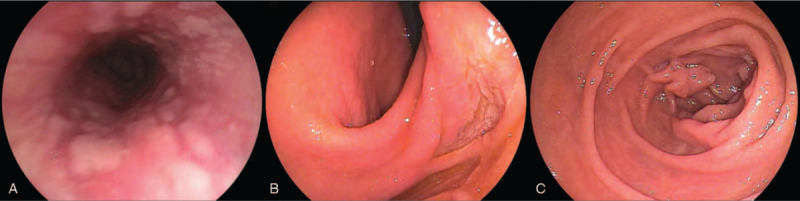
A Extensive mucosal breaks established the diagnosis of reflux esophagitis (class D). B Stomach angle carried both gastric mucosa and intestinal villi. C Papilla was directly exposed in the distal gastric lumen.

Gastroendoscopy played a decisive role in diagnosis, which revealed bile reflux into the esophagus and mucosal breaks involving at least 75% of the esophageal circumference at 32 cm from the incisors. Consistently, we found that the pyloric channel and duodenal bulb were absent, and the duodenal mucosa characterized by villi was adjacent to the lower body of the stomach. Involuntary retrograde passage of bile was draining out from the exposed papilla due to a lack of pyloric constraint (Fig. 2A-C). Apparently, these congenital defects contributed to gastroesophageal reflux disease and caused severe acid regurgitation. Doppler echocardiography revealed both atrial and ventricular septal defects, with sizes of 0.9 cm and 0.36 cm, respectively. We doubted if there was a potential genetic association between multiple variations.

DNA sample preparation (the proband, her parents, and sister), exome capture, and bioinformatics analysis were performed as previously described.^[4]^ Briefly, the preparation of the library of whole exome sequencing (WES) was completed using xGen Exome research panel v1.0 (Integrated DNA Technologies, Coralville, IA, United States) involving the coding sequence of 19,396 genes.^[5]^ Samples were then sequenced on Illumina HiSeq4000 (Illumina, San Diego, CA, United States). However, no putative pathogenic mutation in accordance with the clinical manifestation and modes of inheritance was identified (see Table 1, Supplementary Content, which illustrated details of quality control and WES data of the family).

The patient received proton pump inhibitor and prokinetic treatment for one month and the symptoms apparently improved. She was then transferred to the department of cardiology for the surgical repair of atrial and ventricular septal defects. Besides GERD, recurrent attacks of cholangitis and ulcer formation are foreseeable complications. Fortunately, none of the above occurred in one-year follow-up until the date of writing. Close monitoring will be continued for the potential malignancy risk caused by long-term mucosal damage and unknown genetic variations. Written informed consent for publication of the case was provided by the patient, and the study was approved by the Ethics Committee of Changhai Hospital.

## 3 Discussion

In the present case, the gastroesophageal reflux is similar to the tip of an iceberg. The predisposing aplasia underneath the iceberg is our major concern. The exact reason of ectopic bile duct drainage is not fully known. Several hypotheses have been proposed by Kastinelos et al. to explain the embryologic events.^[6]^ Briefly, the anomalous subdivision of the hepatic diverticulum interferes with the rotation of the stomach. Therefore, the papilla of Vater can aberrantly locate in the stomach wall, duodenal bulb, and pylorus. The true incidence remains unknown because this can be asymptomatic in the entire lifespan of the patient.^[7]^ In several retrospective studies, the frequency of ectopic biliary drainage reported by endoscopic retrograde cholangiopancreatography was 0.11%,^[7]^ 0.2%,^[8]^ and 0.43%.^[9]^ These patients had either cholangitis attacks or choledocholithiasis because of chronic regurgitation of intestinal contents into the biliary tree. Most of them also suffered from ulceration and associated complications including gastric outlet obstruction. Barrett's metaplasia, gastric cancer, and cholangiocarcinoma may develop under constant exposure of reflux material. Prognosis depends on the position of the opening and the reflux pattern. An algorithm of ectopic biliary drainage management has been proposed by Saritas et al. incorporating biliary symptomatology and associated peptic ulcer complications.^[8]^ Low fat diet, acid suppressive medication, prokinetic drugs, and conjugated bile salts can be prescribed to protect the mucosa from biliary injury.^[10]^ Biliary stone formation or stenosis can be managed by endoscopic retrograde cholangiopancreatography. Endoscopic dilation or optional bypass surgery should be considered in cases with gastric outlet obstruction. Fundoplication may be required in well suited GERD cases.^[1]^ Intensive follow-up and endoscopic surveillance are needed due to malignancy risk. In this case, the patient has had an uneventful course so far.

Ectopic papilla of Vater can accompany other congenital anomalies such as congenital fibrous bands in the common bile duct, blind antrum, aberrant pyloric opening, and duodenal atresia.^[11]^ To our knowledge, the rare combination of an absent pylorus and duodenal bulb, congenital blindness of a single eye, and septal defects has not yet been reported. We used a high-throughput sequencing technique to study whether the coexistence of anomalies could be explained by genetic mutation. Unfortunately, no potential pathogenic mutation was identified. However, this conclusion is based on the pathogenic genes that have been already recognized in the known hereditary diseases. Besides, WES is limited to detect genomic imbalances and variation in non-coding sequences. Therefore, unknown gene etiology is expected to be found in the future.

Rare causes of GERD like gastrointestinal abnormalities may be neglected in clinical routine. The diagnostic value of endoscopy is fully demonstrated in such cases. Furthermore, physicians should also be aware of the possibility of involvement of other organ systems. Diagnosis should always be established on the basis of a comprehensive family history as well as personal history, age of onset, clinical presentation, and systemic examinations. Assessment of genetic mutations is feasible when necessary.

## Acknowledgments

None.

## Author contributions

**Conceptualization:** Jia-Yi Ma, Zhao-Shen Li, Liang-Hao Hu.

**Data curation:** Jia-Yi Ma, Dan Wang.

**Formal analysis:** Jia-Yi Ma, Dan Wang.

**Funding acquisition:** Jia-Yi Ma, Dan Wang, Liang-Hao Hu.

**Methodology:** Dan Wang.

**Writing – original draft:** Jia-Yi Ma.

**Writing – review & editing:** Zhao-Shen Li, Liang-Hao Hu.

## Supplementary Material

Supplemental Digital Content
