# Supplementary material for: Severe reflux esophagitis and multiple congenital defects: A case report
Source: Medicine (Baltimore). 2020 Aug 28;99(35):e21758. doi: 10.1097/MD.0000000000021758 (PMC7458243; doi:10.1097/MD.0000000000021758)
Supplement: Supplemental Digital Content [file medi-99-e21758-s001.docx]

Supplementary Table 1: Details of quality control

|  | proband | father | mother | sister |
| --- | --- | --- | --- | --- |
| Targeted gene number | 19396 | 19396 | 19396 | 19396 |
| Length of Targeted regions | 39M | 39M | 39M | 39M |
| QC of the coverage | 92.2% | 91.1% | 91.2% | 91.1% |
| QC of the depth | 71.1 | 90.4 | 105.7 | 89.0 |
| Targeted regions covered by 10X reads | 90.2% | 90.6% | 90.9% | 90.6% |

QC, quality control.
